# Segmentectomy versus wedge resection for radiological solid predominant and low metabolic non-small cell lung cancer

**DOI:** 10.1093/icvts/ivac028

**Published:** 2022-02-07

**Authors:** Atsushi Kagimoto, Yasuhiro Tsutani, Takahiro Mimae, Yoshihiro Miyata, Morihito Okada

**Affiliations:** Department of Surgical Oncology, Hiroshima University, Kasumi, Hiroshima, Japan

**Keywords:** [18F]-fluoro-2-deoxy-D-glucose-positron emission tomography/computed tomography, Deauville criteria, Non-small cell lung cancer, Segmentectomy, Wedge resection

## Abstract

**OBJECTIVES:**

The prognosis of segmentectomy and wedge resection for solid predominant early-stage non-small cell lung cancer with low metabolic activity is unclear.

**METHODS:**

This study aimed to assess patients who underwent segmentectomy or wedge resection with curative intent for clinically node-negative non-small cell lung cancer presenting as a solid predominant tumour (consolidation tumour ratio >50%) with a whole size ≤3 cm and [18F]-fluoro-2-deoxy-D-glucose accumulation weaker than that of the mediastinum tissue (Deauville score, 1 or 2) on positron emission tomography/computed tomography. The cumulative incidence of recurrence (CIR) was compared using the Gray method, and the predictive factor of CIR was analysed using the Fine and Gray method.

**RESULTS:**

Of 140 patients included in this study, 93 (66.4%) underwent segmentectomy and 47 (33.6%) underwent wedge resection. No significant difference in the clinical stage was found between the 2 groups. The CIR was higher with wedge resection than with segmentectomy (*P *=* *0.004). Recurrence after wedge resection was noted in 4 (8.5%) patients, 2 of whom had a recurrent site containing lung parenchyma of the preserved lobe and hilum lymph node, which would have been resected if segmentectomy had been performed. In the multivariable analysis for CIR using inverse probability of treatment weighting and the procedure, wedge resection was a significantly worse predictive factor (hazard ratio, 12.280; *P *=* *0.025).

**CONCLUSIONS:**

Segmentectomy rather than wedge resection should be considered for solid predominant, small-size non-small cell lung cancer even if [18F]-fluoro-2-deoxy-D-glucose accumulation is low.

**Figure:**
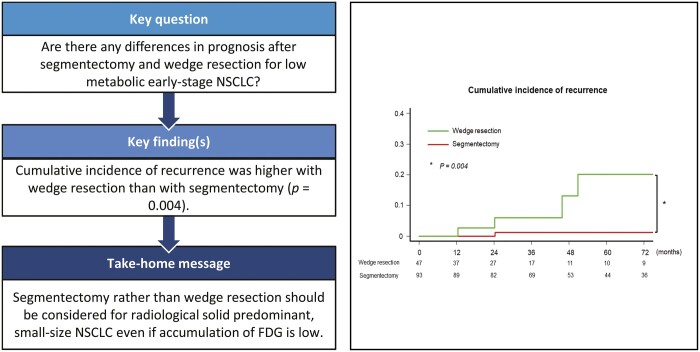

## INTRODUCTION

Lobectomy is the standard procedure in the treatment of non-small cell lung cancer (NSCLC), as supported by the results of a randomized trial [1]. However, some studies present a favourable prognosis with sublobar resection for small size (≤30 mm), clinically node-negative NSCLC [2–5]. Currently, a prospective trial to investigate the feasibility of segmentectomy for early-stage NSCLC is ongoing [6, 7].

In contrast, NSCLC with high [18F]-fluoro-2-deoxy-D-glucose (FDG) accumulation on positron emission tomography (PET)/computed tomography (CT) has invasive characteristics [8], even if its size is small [9]. Based on visual evaluation by FDG-PET/CT, we previously showed that early-stage adenocarcinoma with low FDG accumulation rarely has lymph node metastasis [10] and its prognosis is favourable, irrespective of the procedure [11]. In that study, even in patients with high consolidation tumour ratio (CTR >50%), which is a worse prognostic factor of early-stage NSCLC [12], the incidences of recurrence and lymph node metastasis were low in those with a low FDG accumulation [11]. The frequency of invasive characteristics, such as lymphatic invasion, vascular invasion and pleural invasion, were also low in patients with low FDG accumulation [11]. Therefore, this study was conducted based on the hypothesis that wedge resection may have a prognosis similar to that of segmentectomy for NSCLC with low FDG accumulation, even if its CTR is high.

## PATIENTS AND METHODS

### Ethics statement

This retrospective study was approved by the Institutional Review Board of Hiroshima University Hospital, Hiroshima, Japan (approval number, E-1216). The requirement for informed consent from individual patients was waived because it was a retrospective study.

### Patients

**Figure 1:**
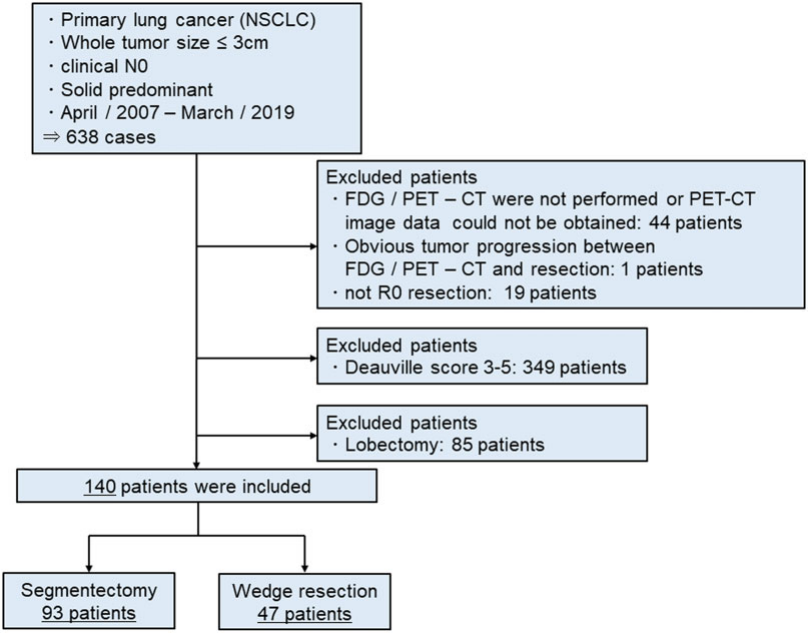
Flow chart for the study patients. Patients who underwent segmentectomy or wedge resection for non-small cell lung cancer that presented as a solid component predominant tumour (CTR >50%) on preoperative computed tomography with a whole tumour size of ≤3 cm and a low accumulation of [18F]-fluoro-2-deoxy-D-glucose in preoperative [18F]-fluoro-2-deoxy-D-glucose-positron emission tomography/computed tomography (Deauville score 1 or 2). A total of 140 patients were included in this study. CT: computed tomography; CTR: consolidation tumour ratio; FDG: [18F]-fluoro-2-deoxy-D-glucose; NSCLC: non-small cell lung cancer; PET: positron emission tomography.

We analysed the clinicopathological data and prognosis of patients who underwent segmentectomy or wedge resection with curative intent for NSCLC that presented as a node-negative, solid component predominant tumour (CTR >50%) with a whole tumour size ≤3 cm on preoperative CT and had a low FDG accumulation on preoperative FDG-PET/CT between April 2007 and March 2019 at Hiroshima University Hospital. FDG accumulation on PET/CT was evaluated using the Deauville criteria, a visual 5-point scale evaluating FDG-PET/CT characteristics [12]. Based on the results of our previous study, a Deauville score of 1–2 was considered low accumulation, whereas a score of 3–5 was considered high accumulation [10, 11]. Supplementary Material, Table S1 shows additional details of the Deauville criteria. The exclusion criteria were as follows: previous induction therapy; small cell lung cancer or carcinoid; whole tumour size >3 cm or high FDG accumulation on PET/CT (Deauville score 3–5); and suspicious lymph node metastasis (enlargement >1 cm in preoperative CT or significant FDG accumulation on PET/CT) preoperatively. Patients without complete resection and those with obvious tumour progression after FDG-PET/CT were also excluded from this study. Figure 1 presents a flowchart of patient selection. The primary endpoint of this study was cumulative incidence of recurrence (CIR).

### Preoperative examination

Preoperative evaluations, including chest CT, whole-body FDG-PET/CT, brain magnetic resonance imaging and pulmonary function tests, were performed to determine the clinical stage and treatment strategies. Staging was based on the TNM Classification for Lung Cancer, 8th edition, for malignant tumours [13].

### Surgical procedures

Surgery was performed with hybrid video-assisted thoracic surgery [14]. In our institution, sublobar resection is performed for patients with tumour <2 cm, those with tumour with low metabolic activity, and patients considered intolerant to lobectomy. The decision regarding which sublobar procedure to perform (i.e. segmentectomy or wedge resection) is usually based on a combination of patient performance status, tumour characteristics and surgeon preference. Wedge resection is preferred for small, pleural-based tumours, particularly for patients with poor performance status and comorbidities, whereas segmentectomy is usually required for large tumours that settle within a resectable anatomical segment.

### Histologic and pathologic evaluations

The determination of pathologic stages was based on the TNM Classification for Lung Cancer, 8th edition, for malignant tumours [13]. Determination of the histologic subtype was based on the World Health Organization classification [15]. The diagnosis of lymphatic invasion was based on pathologic examination using immunostaining for D2-40 to clarify the location of the lymphatic ducts. The presence of vascular invasion and pleural invasion was evaluated using elastic van Gieson staining to determine tumour invasion above the elastic layer of the vessels and visceral pleura.

### Follow-up evaluation

Postoperative follow-up procedures, including physical examination and chest CT every 6 months, were performed for 5–10 years after surgical resection. Recurrence was determined based on radiographic features or histological evidence.

### Statistical analysis

The results were presented as medians and interquartile ranges for continuous variables and numbers and percentages for categorical variables. Continuous variables that were normally distributed were analysed using Student’s *t*-test. Continuous variables that were non-normally distributed were analysed using Wilcoxon’s rank-sum test. Pairwise analysis was used for analysis that contains missing data. McNemar’s test for categorical variables and paired *t*-tests for continuous variables were used to analyse propensity-matched patient pairs. Prognosis was analysed using competing risk analysis. The risk of recurrence (defined as CIR), which is the period from surgery to recurrence, was the main outcome of this study and estimated using a cumulative incidence function that accounted for mortality without recurrence as a competing event. The risk of death without recurrence [defined as cumulative incidence of death without recurrence (CIDWR), which is the period from surgery to death without recurrence] was also estimated using a cumulative incidence function that accounted for recurrence as a competing event. Patients were censored if they were alive and without recurrence at the time of the last follow-up. Differences in CIR and CIDWR between groups were assessed using the Gray method.

Propensity scores were estimated using a logistic regression model that included solid component size (continuous value), CTR (continuous value) and histology (adenocarcinoma or non-adenocarcinoma). Matching cohorts were formed using this propensity score; segmentectomy and lobectomy group pairs with an equivalent propensity score were selected by a 1-to-1 match with a calliper width of 0.2 of standard deviation.

Inverse probability of treatment weighting (IPTW) based on propensity scores was also used to adjust for differences in covariates between both groups, and multivariable analysis for CIR was performed using the IPTW and surgical method to investigate whether the surgical procedure affected prognosis using the Fine and Gray methods.

All statistical analyses were performed using EZR version 1.51 (Saitama Medical Center, Jichi Medical University, Saitama, Japan) [16], which is a graphical user interface for R (The R Foundation for Statistical Computing, Vienna, Austria).

## RESULTS

A total of 140 patients were included in this study. The distribution of patients is shown in Supplementary Material, Fig. S1. Patient characteristics are presented in Table 1. Of the 140 study patients, 93 (66.4%) underwent segmentectomy and 47 (33.6%) underwent wedge resection. Although the whole tumour size was larger in patients who underwent segmentectomy than in those who underwent wedge resection (*P *=* *0.030), no difference was noted in the solid component size (*P *=* *0.533) and clinical stage (*P *=* *0.536). The incidence of adenocarcinoma was higher in patients who underwent segmentectomy (*P *=* *0.035). There was no difference in the pathological stage between patients who underwent segmentectomy and those who underwent wedge resection (*P *=* *0.826). Eight patients had pathological stage IB. The diagnosis of stage IB was based on pleural invasion, not tumour diameter.

**Table 1:** Patient characteristics

Variables	Segmentectomy *n* = 93 (66.4%)	Wedge resection *n* = 47 (33.6%)	*P*-value
Age (median) (IQR)	69 (64–74)	73 (65–80)	0.045
Sex (%)			0.370
Male	50 (53.8%)	29 (61.7%)	
Female	43 (46.2%)	18 (38.3%)	
CEA (mg/dl)	2.5 (1.4–4.1)	3.0 (1.8–4.7)	0.114
Tumour size			
Whole tumour size (mm) (median) (IQR)	15 (12–19)	13 (10–17)	0.030
Solid component size (mm) (median) (IQR)	12 (9–15)	12 (9–14)	0.533
CTR (median) (IQR)	0.80 (0.62–1.00)	1.00 (0.80–1.00)	0.013
Pure solid (CTR 1.0)	38 (40.9%)	26 (55.3%)	0.105
Deauville score			0.756
1	24 (25.8%)	11 (23.4%)	
2	69 (74.2%)	36 (76.6%)	
SUVmax	1.2 (0.8–1.6)	1.2 (0.8–1.5)	0.995
Clinical stage (%)			0.536
IA1	31 (33.3%)	18 (38.3%)	
IA2	59 (63.4%	26 (55.3%)	
IA3	3 (3.3%)	3 (6.4%)	
Extent of lymph node dissection			<0.001
0	0 (0%)	45 (95.7%)	
1b	13 (14.0%)	0 (0%)	
2a-1	80 (86%)	0 (0%)	
Sampling of mediastinal lymph node	0 (0%)	2 (1.4%)	
Number of resected lymph nodes	5 (3–8)	0 (0–0)	<0.001
Histological subtype (%)			0.035
Adenocarcinoma	86 (92.5%)	41 (87.2%)	
Predominant subtype of adenocarcinoma			0.260
Lepidic	27 (31.4%)	16 (39.0%)	
Papillary	53 (61.6%)	18 (43.9%)	
Acinar	3 (3.5%)	2 (4.9%)	
Solid	2 (2.3%)	2 (4.9%)	
Micropapillary	0 (0%)	1 (2.4%)	
Invasive mucinous adenocarcinoma	1 (1.2%)	2 (4.9%)	
Squamous cell carcinoma	7 (7.5%)	3 (6.4%)	
Adenosquamous carcinoma	0 (0%)	3 (6.4%)	
LY	4 (4.3%)	7 (14.9%)	0.034
V	7 (7.5%)	9 (19.2%)	0.048
PL	4 (4.3%)	5 (10.6%)	0.162
EGFR mutation (among adenocarcinoma)			
Positive	22 (42.3%)	6 (31.6%)	0.408
Negative	30 (57.7%)	13 (68.4%)	
Unknown	34	22	
STAS (among adenocarcinoma)			
Positive	26 (32.1%)	19 (54.3%)	0.025
Negative	55 (67.9%)	16 (45.7%)	
Unknown	5	6	
Pathologic stage (%)			0.826
0	11 (11.9%)	7 (14.9%)	
IA1	38 (40.9%)	19 (40.4%)	
IA2	34 (36.6%)	13 (27.7%)	
IA3	4 (4.3%)	3 (6.4%)	
IB	4 (4.3%)	4 (8.5%)	
IIB	2 (2.2%)	1 (2.1%)	
Resection margin (mm)	15 (8–20)	10 (8–14)	0.001
Lymph node metastasis	1 (1.1%)	0 (0%)	0.365
Prognosis			
Recurrence	1 (1.1%)	4 (8.5%)	0.030
Death from any cause	4 (4.3%)	7 (14.9%)	0.034
Death from lung cancer	0 (0%)	2 (4.3%)	0.035

CEA: carcinoembryonic antigen; CTR: consolidation tumour ratio; EGFR: epidermal growth factor receptor; IQR: interquartile range; LY: lymphatic invasion; PL: pleural invasion; STAS: spread through air spaces.; SUV: maximum standardized uptake value; V: vascular invasion.

**Figure 2:**
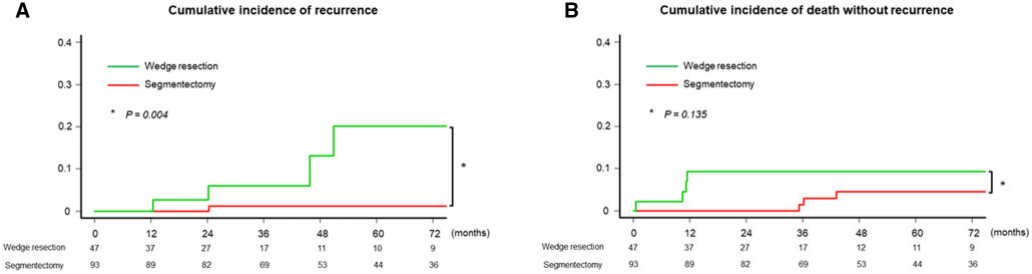
(**A**) Cumulative incidence of recurrence (CIR) after segmentectomy and wedge resection. CIR was higher in patients who underwent wedge resection [5-year CIR rate 20.1%, 95% confidence interval (CI) 5.1–42.2%) than in those who underwent segmentectomy (5-year CIR rate 1.2%, 95% CI 0.1–5.9%, *P *=* *0.004). (**B**) No significant difference was found in cumulative incidence of death without recurrence (CIDWR) between patients who underwent wedge resection (5-year CIDWR rate 9.3%, 95% CI 2.9–20.3%) and segmentectomy (5-year CIDWR rate 4.5%, 95% CI 1.2–11.6%, *P *=* *0.135).

The median follow-up duration was 43 months (interquartile range, 24–75 months). The CIR was significantly higher in patients who underwent wedge resection [5-year CIR rate 20.1%, 95% confidence interval (CI) 5.1–42.2%] than in those who underwent segmentectomy (5-year CIR rate 1.2%, 95% CI 0.1–5.9%, *P *=* *0.004; Fig. 2A]. No significant difference in CIDWR was noted between patients who underwent wedge resection (5-year CIDWR rate 9.3%, 95% CI 2.9–20.3%) and those who underwent segmentectomy (5-year CIDWR rate 4.5%, 95% CI 1.2–11.6%, *P *=* *0.135; Fig. 2B).

In the analysis of patients with a whole tumour size ≤2 cm, CIR was significantly higher in patients who underwent wedge resection (5-year CIR rate 20.2%, 95% CI 5.2–42.3%) than in those who underwent segmentectomy (5-year CIR rate 0%, *P *<* *0.001; Supplementary Material, Fig. S2A). No significant difference was noted in CIDWR between patients who underwent wedge resection (5-year CIDWR rate 9.8%, 95% CI 3.1–21.3%) and those who underwent segmentectomy (5-year CIDWR rate 5.0%, 95% CI 1.3–12.7%, *P *=* *0.160; Supplementary Material, Fig. S2B).

**Figure 3:**
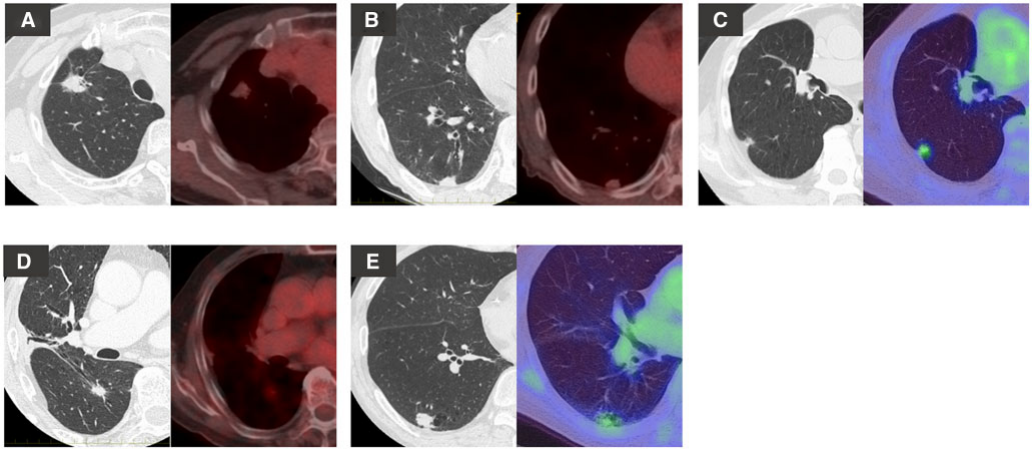
Preoperative computed tomography and [18F]-fluoro-2-deoxy-D-glucose-positron emission tomography/computed tomography images of (**A**) Case 1, (**B**) Case 2, (**C**) Case 3, (**D**) Case 4 and (**E**) Case 5 as presented in Table 2.

The characteristics and preoperative image of patients with relapse are shown in Table 2 and Fig. 3, respectively. No recurrences were noted in patients with Deauville score 1; however, relapse occurred in 4 patients (8.5%) after wedge resection and 1 (1.1%) patient after segmentectomy. Among patients who underwent wedge resection, 2 patients had recurrence only at the lesion site where the tissue would not have been resected even if segmentectomy were performed (Case 1, pleural dissemination; Case 4, bone metastasis). In contrast, relapse occurred in 2 other patients, in whom the tissue may have been resected if segmentectomy were performed (Case 2, hilar and mediastinum lymph node; Case 3, lung at preserved lobe).

**Table 2:** Characteristics of patients who had recurrence after resection

Case number Procedure (lymph node dissection)	Age Sex	Clinical stage (total/solid component size) Deauville score	Pathologic stage (total/invasive size) (LY, V, PL) (STAS)	Resection margin	Histology	Recurrent site	Prognosis
Case 1 Wedge resection (none)	89F	cT1cN0M0 24 mm/21 mm Deauville score 2	pT1cN0M0 24 mm/21 mm (LY0, V0, PL0) (STAS negative)	2 mm	Adenocarcinoma (Papillary predominant)	Dissemination	Death from lung cancer
Case 2 Wedge resection (none)	86M	cT1bN0M0 15 mm/15 mm Deauville score 2	pT1bN0M0 20 mm/20 mm (LY0, V1, PL0) (STAS unknown)	15 mm	Adenosquamous carcinoma	Hilar lymph node, mediastinum lymph node, supraclavicular lymph node, liver	Alive
Case 3 Wedge resection (none)	75M	cT1aN0M0 9 mm/9 mm Deauville score 2	pT3N0M0 (Parietal pleural invasion 9 mm/9 mm (LY1, V1, PL1) (STAS positive)	5 mm	Adenosquamous carcinoma	Lung (preserved lobe)	Alive
Case 4 Wedge resection (none)	84M	cT1bN0M0 17 mm/12 mm Deauville score 2	pT2aN0M0 20 mm/10 mm (LY1, V1, PL0) (STAS unknown)	7 mm	Adenocarcinoma (Papillary predominant)	Multiple bone	Death from lung cancer
Case 5 Segmentectomy (ND 2a-1)	83M	cT1cN0M0 23 mm/23 mm Deauville score 2	pT1cN0M0 25 mm/25 mm (LY0, V1, PL1) (STAS positive)	15 mm	Adenocarcinoma (Papillary predominant)	Mediastinal lymph node, multiple lung (ipsilateral, not resected lobe)	Alive

F: female; LY: lymphatic invasion; M: male; PL: pleural invasion; STAS: spread through air spaces; V: vascular invasion.

Supplementary Material, Table S2 shows the characteristics of the matched cohort. The CIR was higher in patients who underwent wedge resection (5-year CIR rate 12.2%, 95% CI 1.4–35.5%) than in those who underwent segmentectomy (5-year CIR rate 0%; Supplementary Material, Fig. S3A). The 5-year CIDWR rates were 9.3% (95% CI 2.3–22.5%) and 4.2% (95% CI 0.3–18.0%) in patients who underwent wedge resection and segmentectomy, respectively.

In the multivariable analysis for CIR using IPTW and procedure (characteristics of IPTW cohort are shown in Supplementary Material, Table S3), wedge resection was a significantly worse predictive factor of CIR (hazard ratio 12.980, 95% CI 1.601–105.300, *P *=* *0.016, Table 3).

**Table 3:** Inverse probability of treatment weighting-adjusted multivariable analysis for cumulative incidence of recurrence

Variables	HR (95% CI)	*P*-value
Procedure (wedge resection/segmentectomy [ref])	12.280 (1.374–109.70)	0.025

CI: confidence interval; HR: hazard ratio.

## DISCUSSION

In this study, CIR after wedge resection was worse than that after segmentectomy, and no significant difference was noted between procedures in CIDWR. In the multivariable analysis, wedge resection was a significantly worse predictive factor of recurrence. These results indicate that, even for a tumour with low metabolic activity, segmentectomy should be considered first as a sublobar resection for NSCLC that presents as a solid predominant (CTR > 50%) tumour.

In this study, CIR was the primary endpoint. The background characteristics differed between patients who underwent segmentectomy and those who underwent wedge resection. In deciding the surgical procedure, selection bias may have been present. Recurrence-free survival and overall survival are often used as endpoints of this type of retrospective study. However, recurrence and death from causes other than lung cancer are treated as events in the analysis using recurrence-free survival. In the analysis using overall survival, all deaths are equally treated as events, regardless of cause of death. In the analysis of early-stage NSCLC, the incidence of death from causes other than lung cancer is high; therefore, CIR was used as a primary endpoint to assess the oncologic effects between segmentectomy and wedge resection, considering the competing risk such as non-cancer events.

It is already known that wedge resection can provide sufficient prognosis for NSCLC with low malignancy, such as a tumour with low CTR (≤50%) [4, 17]. Regarding early-stage NSCLC with high CTR, a randomized trial is ongoing, and segmentectomy can be a standard treatment strategy for NSCLC with whole tumour size ≤2 cm. To the best of our knowledge, no randomized study has been conducted to compare segmentectomy and lobectomy for NSCLC with whole tumour size ≤3 cm. However, the retrospective study shows that prognosis after segmentectomy is equivalent to that after lobectomy, including NSCLC with whole tumour size ≤3 cm [18–20]. Therefore, segmentectomy may become the standard treatment for NSCLC with whole tumour size ≤3 cm. Generally, as a sublobar resection, segmentectomy is superior to wedge resection in terms of postoperative prognosis [21]. In contrast, it is also known that FDG-PET/CT is useful in predicting invasive characteristics of NSCLC. Therefore, we hypothesized that wedge resection can provide sufficient prognosis in patients with a tumour that has low metabolic activity, even if CTR is high.

We reported that the Deauville score is a significant predictive factor of lymph node metastasis [10] and prognosis [11] for early-stage lung adenocarcinoma; in our study, no difference was found in the prognosis after lobectomy, segmentectomy and wedge resection in patients with low Deauville score [11]. Conversely, CTR is also a worse prognostic factor of early-stage NSCLC [22]; therefore, we analysed the incidence of lymph node metastasis and recurrence by classifying patients as having CTR ≤50% or CTR >50%. In that analysis, most patients with CTR ≤50% had a Deauville score of 1 or 2, and no recurrence and lymph node metastasis were noted. This result is consistent with the results of previous studies on CTR, and it was important to investigate the relationship between prognosis, procedure and FDG accumulation in patients with a high CTR. Because it was already known that NSCLC with high FDG accumulation develop lymph node metastasis even if it is clinically node-negative, patients with a high Deauville score were excluded from this study that compared segmentectomy with lymph node dissection and wedge resection because usually lymph node dissection is not performed. Initially, we expected that the prognosis would be the same regardless of whether we performed segmentectomy or wedge resection. However, the results were unexpected. In our study, 4 patients had recurrence after wedge resection. Of these patients, 2 had relapse with a site outside the area to be resected if segmentectomy were performed. Recurrence in these patients may have been inevitable, even if they had undergone segmentectomy. Recurrence for 2 other patients could have been prevented because their recurrent site contained lung parenchyma of preserved lobe and hilum lymph node, which would have been resected if segmentectomy were performed. The merit of segmentectomy compared with wedge resection is that an adequate surgical margin can be obtained for a centrally located tumour that is difficult to treat by wedge resection. Moreover, hilar lymph nodes may be dissected by segmentectomy but not by wedge resection. These factors probably contribute to better prognosis after segmentectomy.

### Limitations

This study has several limitations. First, this study is from a single institution, and the number of study patients was restricted. Particularly, wedge resection is not a standard procedure for NSCLC with high CTR; thus, the number of patients who underwent wedge resection was small. Hazard ratios in multivariable analysis are large and 95% CIs in prognostic analysis and multivariable analysis are wide. It is possible that collinearity, small sample size or low rate of recurrence affected results of our study. Second, because this study design is retrospective and the decision on the procedure is finally influenced by the preference of the attending surgeon and patients, some bias may have affected the results. Although the reason was unclear, the proportion of patients who underwent segmentectomy and wedge resection was different for each year. To confirm the results of this study, a prospective study is needed. However, wedge resection was found to be a worse prognostic factor of CIR in the multivariable analysis. This finding indicates that segmentectomy should be considered first. Therefore, the significance of this study is profound because no other study has compared wedge resection and segmentectomy for a solid predominant tumour with low metabolic activity.

## CONCLUSION

In patients with NSCLC presenting with a solid predominant tumour on preoperative CT with a whole tumour size ≤3 cm, prognosis after wedge resection was worse than that after segmentectomy, even if FDG accumulation was low. Regarding the treatment strategy for early-stage NSCLC with high CTR, segmentectomy should be considered first even if FDG accumulation is low.

## SUPPLEMENTARY MATERIAL

Supplementary material is available at *ICVTS* online.

## Funding

This work was supported by Japan Society for the Promotion of Science KAKENHI (Grant Number JP20K17749).

**Conflict of interest:** none declared. 

## Data availability statement

The data underlying this article are available in the article and in its online supplementary material.

## Author contributions

**Atsushi Kagimoto:** Conceptualization; Data curation; Formal analysis; Writing—original draft. **Yasuhiro Tsutani:** Conceptualization; Data curation; Supervision; Writing—review & editing. **Takahiro Mimae:** Data curation; Writing—review & editing. **Yoshihiro Miyata:** Data curation; Writing—review & editing. **Morihito Okada:** Supervision.

## Reviewer information

Interactive CardioVascular and Thoracic Surgery thanks René Horsleben Petersen, Gonzalo Varela and the other anonymous reviewers for their contribution to the peer review process of this article.

## Supplementary Material

ivac028_Supplementary_Data

## ABBREVIATIONS

CI: Confidence interval
CIDWR: Cumulative incidence of death without recurrence
CIR: Cumulative incidence of recurrence
CT: Computed tomography
CTR: Consolidation tumour ratio
FDG: [18F]-ﬂuoro-2-deoxy-D-glucose
IPTW: Inverse probability of treatment weighting
NSCLC: Non-small cell lung cancer
PET: Positron emission tomography
