# Supplementary material for: Segmentectomy versus wedge resection for radiological solid predominant and low metabolic non-small cell lung cancer
Source: Interact Cardiovasc Thorac Surg. 2022 Feb 7;34(5):814–21. doi: 10.1093/icvts/ivac028 (PMC9070489; doi:10.1093/icvts/ivac028)
Supplement: ivac028_Supplementary_Data [file ivac028_supplementary_data.zip › 20211231Supplemental Figure legend.docx]

**Supplemental Figure legend**

**Supplementary Materials, Figure S1**

Distribution of included patients.

**Supplementary Materials, Figure S2**

Analysis of patients with a whole tumor size ≤2 cm. (A) Cumulative incidence of recurrence (CIR) was significantly higher in patients who underwent wedge resection (5-year CIR rate, 20.2%; 95% confidence interval [CI], 5.2%–42.3%) than in those who underwent segmentectomy (5-year CIR rate, 0%; *p* < 0.001). No significant difference was noted in the cumulative incidence of death without recurrence (CIDWR) between patients who underwent wedge resection (5-year CIDWR rate, 9.8%; 95% CI 3.1%–21.3%) and those who underwent segmentectomy (5-year CIDWR rate, 5.0%; 95% CI, 1.3%–12.7%; *p* = 0.160).

**Supplementary Materials, Figure S3**

CIR and CIDWR of the matched cohort. (A) CIR was higher in patients who underwent wedge resection (5-year CIR rate, 12.2%; 95% CI, 1.40%–35.5%) than in those who underwent segmentectomy (5-year CIR rate, 0%). (B) The 5-year CIDWR rates were 9.3% (95% CI, 2.3%–22.5%) and 4.2% (95% CI, 0.3%–18.0%) in patients who underwent wedge resection and those who underwent segmentectomy, respectively.
