# Supplementary material for: Segmentectomy versus wedge resection for radiological solid predominant and low metabolic non-small cell lung cancer
Source: Interact Cardiovasc Thorac Surg. 2022 Feb 7;34(5):814–21. doi: 10.1093/icvts/ivac028 (PMC9070489; doi:10.1093/icvts/ivac028)
Supplement: ivac028_Supplementary_Data [file ivac028_supplementary_data.zip › Supplementary Materials Table S1.docx]

| Deauville Score | Result of FDG-PET / CT |
| --- | --- |
| 1 | No uptake stronger than background lung field |
| 2 | Uptake weaker or same to mediastinum |
| 3 | Uptake stronger than mediastinum, but weaker or same to liver |
| 4 | Uptake moderately stronger than liver at any site |
| 5 | Uptake markedly stronger than liver at any site |
| FDG, [18F]-ﬂuoro-2-deoxy-D-glucose; PET, positron emission tomography; CT, computed tomography | |

**Supplementary Materials, Table S1. Scoring system of Deauville criteria**
