# Supplementary material for: Segmentectomy versus wedge resection for radiological solid predominant and low metabolic non-small cell lung cancer
Source: Interact Cardiovasc Thorac Surg. 2022 Feb 7;34(5):814–21. doi: 10.1093/icvts/ivac028 (PMC9070489; doi:10.1093/icvts/ivac028)
Supplement: ivac028_Supplementary_Data [file ivac028_supplementary_data.zip › Supplementary Materials Table S2.docx]

**Supplementary Materials, Table S2. Patient characteristics of matched cohort**

| Variables | Segmentectomy  n = 35 | Wedge resection  n = 35 | *p* value | SMD |
| --- | --- | --- | --- | --- |
| Age (median) (IQR) | 68 (63–73) | 73 (64–79) | 0.094 |  |
| Sex　(%) |  |  | 0.606 |  |
| Male | 25 (71.4%) | 23 (65.7%) |  |  |
| Female | 10 (28.6%) | 12 (34.3%) |  |  |
| CEA (mg/dl) | 2.6 (1.4–4.9) | 2.8 (1.6–4.6) | 0.661 |  |
| Tumor size |  |  |  |  |
| Whole tumor size (mm) (median) (IQR) | 13 (12–18) | 12 (10–16) | 0.085 |  |
| Solid component size (mm) (median) (IQR) | 11 (10–14) | 11 (9–13) | 0.492 | 0.165 |
| CTR (median) (IQR) | 0.9 (0.6–1.0) | 1.0 (0.8–1.0) | 0.155 | -0.344 |
| Pure solid (CTR 1.0) | 17 (48.6%) | 20 (57.1%) | 0.472 |  |
| Deauville score |  |  | 0.445 |  |
| 1 | 13 (37.1%) | 10 (28.6%) |  |  |
| 2 | 22 (62.9%) | 25 (71.4%) |  |  |
| SUVmax | 1.2 (0.8–1.4) | 1.2 (0.8–1.5) | 0.425 |  |
| Clinical Stage (%) |  |  | 0.454 |  |
| IA1 | 11 (31.4%) | 24 (68.6%) |  |  |
| IA2 | 14 (40.0%) | 21 (60.0%) |  |  |
| Extent of lymph node dissection |  |  | < 0.001 |  |
| 0 | 0 (0%) | 33 (94.3%) |  |  |
| 1b | 4 (11.4%) | 0 (0%) |  |  |
| 2a-1 | 31 (88.6%) | 0 (0%) |  |  |
| Sampling of mediastinal lymph node | 0 (0%) | 2 (5.7%) |  |  |
| Number of resected lymph nodes | 5 (3–7) | 0 (0–0) | < 0.001 |  |
| Histological subtype (%) |  |  | 0.450 | 0.180 |
| Adenocarcinoma | 32 (91.4%) | 30 (85.7%) |  |  |
| Squamous cell carcinoma | 3 (8.6%) | 4 (11.4%) |  |  |
| Adenosquamous carcinoma | 0 (0%) | 1 (2.9%) |  |  |
| Predominant subtype of adenocarcinoma |  |  | 0.385 |  |
| Lepidic | 13 (40.6%) | 14 (46.7%) |  |  |
| Papillary | 16 (50.0%) | 11 (36.7%) |  |  |
| Acinar | 3 (9.4%) | 2 (6.7%) |  |  |
| Solid | 0 (0%) | 1 (3.3%) |  |  |
| Micropapillary | 0 (0%) | 1 (3.3%) |  |  |
| Invasive mucinous adenocarcinoma | 0 (0%) | 1 (3.3%) |  |  |
| LY | 2 (5.7%) | 3 (8.6%) | 0.642 |  |
| V | 2 (5.7%) | 7 (20.0%) | 0.067 |  |
| PL | 3 (8.6%) | 3 (8.6%) | 1.000 |  |
| EGFR mutation (among adenocarcinoma) |  |  |  |  |
| Positive | 6 (33.3%) | 2 (16.7%) | 0.302 |  |
| Negative | 12 (66.7%) | 10 (83.3%) |  |  |
| Unknown | 14 | 18 |  |  |
| STAS (among adenocarcinoma) |  |  |  |  |
| Positive | 7 (22.6%) | 14 (56.0%) | 0.010 |  |
| Negative | 24 (77.4%) | 11 (44.0%) |  |  |
| Unknown | 1 | 5 |  |  |
| Pathologic stage (%) |  |  | 0.988 |  |
| 0 | 6 (17.1%) | 6 (17.1%) |  |  |
| IA1 | 16 (45.7%) | 14 (40.0%) |  |  |
| IA2 | 9 (25.7%) | 11 (31.4%) |  |  |
| IA3 | 1 (2.9%) | 1 (2.9%) |  |  |
| IB | 3 (8.6%) | 3 (8.6%) |  |  |
| Resection margin (mm) | 20 (10–30) | 10 (8–13) | < 0.001 |  |
| Lymph node metastasis | 0 (0%) | 0 (0%) | NA |  |
| Prognosis |  |  |  |  |
| Recurrence | 0 (0%) | 2 (5.7%) | 0.092 |  |
| Death from any cause | 0 (0%) | 1 (1.4%) | 0.236 |  |
| Death from lung cancer | 2 (5.7%) | 4 (11.4%) | 0.389 |  |

IQR, interquartile range; SMD, standardized mean difference; CEA, carcinoembryonic antigen; CTR, consolidation tumor ratio; SUV, maximum standardized uptake value; LY, lymphatic invasion; V, vascular invasion; PL, pleural invasion; EGFR, epidermal growth factor receptor; STAS, spread through air spaces.
