# Supplementary material for: Segmentectomy versus wedge resection for radiological solid predominant and low metabolic non-small cell lung cancer
Source: Interact Cardiovasc Thorac Surg. 2022 Feb 7;34(5):814–21. doi: 10.1093/icvts/ivac028 (PMC9070489; doi:10.1093/icvts/ivac028)
Supplement: ivac028_Supplementary_Data [file ivac028_supplementary_data.zip › Supplementary Materials Table S3.docx]

**Supplementary Materials, Table S3. Patient characteristics of inverse probability of treatment weighting (IPTW) cohort**

| Variables | Segmentectomy  n = 92.5 | Wedge resection  n = 47.8 | *p* value | SMD |
| --- | --- | --- | --- | --- |
| Age (median) (IQR) | 68.48 (63.05-74.00) | 73.00 (66.16-80.83) | 0.009 |  |
| Sex　(%) |  |  | 0.737 |  |
| Male | 51.9 (56.2%) | 28.4 (59.3%) |  |  |
| Female | 40.6 (43.8%) | 19.4 (40.7%) |  |  |
| CEA (mg/dl) | 2.49 (1.40-4.03) | 3.23 (1.72-4.75) | 0.123 |  |
| Tumor size |  |  |  |  |
| Whole tumor size (mm) (median) (IQR) | 14.16 (12-18) | 3.23 (1.72-4.75) | 0.840 |  |
| Solid component size (mm) (median) (IQR) | 12 (9.48-14.94) | 11.80 (9.68-14.00) | 0.923 | 0.026 |
| CTR (median) (IQR) | 0.9 (0.65-1.00) | 0.84 (0.70-1.00) | 0.997 | 0.088 |
| Pure solid (CTR 1.0) | 43.4 (46.9%) | 19.9 (41.7%) | 0.568 |  |
| Deauville score |  |  | 0.679 |  |
| 1 | 24.0 (26.0%) | 10.8 (22.6%) |  |  |
| 2 | 68.5 (74.0%) | 37.0 (77.4%) |  |  |
| SUVmax | 1.20 (0.00-3.10) | 1.11 (0.00-3.30) | 0.934 |  |
| Clinical Stage (%) |  |  | 0.372 |  |
| IA1 | 30.7 (33.2%) | 13.9 (29.0%) |  |  |
| IA2 | 59.2 (64.0%) | 29.9 (62.5%) |  |  |
| IA3 | 2.6 (2.8%) | 4.1 (8.5%) |  |  |
| Extent of lymph node dissection |  |  | < 0.001 |  |
| 0 | 0 (0%) | 45.8 (95.7%) |  |  |
| 1b | 12.6 (13.6%) | 0 (0%) |  |  |
| 2a-1 | 79.9 (86.4%) | 0 (0%) |  |  |
| Sampling of mediastinal lymph node | 0 (0%) | 2 (4.3%) |  |  |
| Number of resected lymph nodes | 5.00 (3.05-8.00) | 0 (0-0) | < 0.001 |  |
| Histological subtype (%) |  |  | 0.942 | 0.012 |
| Adenocarcinoma | 84 (90.8%) | 43.6 (91.2%) |  |  |
| Squamous cell carcinoma | 8.5 (9.2%) | 2.0 (4.1) |  |  |
| Adenosquamous carcinoma | 0 (0%) | 2.2 (4.7%) |  |  |
| Predominant subtype of adenocarcinoma |  |  | 0.139 |  |
| Lepidic | 24.0 (25.9%) | 17.5 (36.7%) |  |  |
| Papillary | 53.4 (57.7%) | 18.7 (39.2%) |  |  |
| Acinar | 3.4 (3.7%) | 1.3 (2.8%) |  |  |
| Solid | 2.2 (2.4%) | 1.4 (2.9%) |  |  |
| Micropapillary | 0 (0%) | 0.7 (1.5%) |  |  |
| Invasive mucinous adenocarcinoma | 1.0 (1.1%) | 3.9 (8.2%) |  |  |
| LY | 4.8 (5.1%) | 6.3 (13.2%) | 0.115 |  |
| V | 7.5 (8.1%) | 8.4 (17.5%) | 0.114 |  |
| PL | 4.6 (5.0%) | 4.3 (9.0%) | 0.365 |  |
| EGFR mutation (among adenocarcinoma) |  |  | 0.329 |  |
| Positive | 21.1 (22.8%) | 7.8 (16.3%) |  |  |
| Negative | 31.9 (34.5%) | 12.8 (26.7%) |  |  |
| Unknown | 39.5 (42.7%) | 27.3 (57.0%) |  |  |
| STAS (among adenocarcinoma) |  |  | 0.271 |  |
| Positive | 27.0 (29.2%) | 16.9 (35.4%) |  |  |
| Negative | 51.7 (55.9%) | 19.7 (41.2%) |  |  |
| Unknown | 13.8 (14.9%) | 11.2 (23.4%) |  |  |
| Pathologic stage (%) |  |  | 0.573 |  |
| 0 | 10.4 (11.2%) | 6.9 (14.3%) |  |  |
| IA1 | 38.3 (41.4%) | 17.6 (36.8%) |  |  |
| IA2 | 33.4 (36.1%) | 13.5 (28.3%) |  |  |
| IA3 | 3.8 (4.2%) | 5.5 (11.5%) |  |  |
| IB | 4.6 (5.0%) | 3.7 (7.7%) |  |  |
| IIB | 2.1 (2.2%) | 0.6 (1.3%) |  |  |
| Resection margin (mm) | 15 (8.92-20.0) | 10.0 (7.15-13.32) | 0.001 |  |
| Lymph node metastasis | 1.2 (1.3%) | 0 (0%) | 0.470 |  |
| Prognosis |  |  |  |  |
| Recurrence | 0.9 (0.9%) | 4.5 (9.4%) | 0.011 |  |
| Death from any cause | 3.9 (4.2%) | 7.3 (15.2%) | 0.031 |  |
| Death from lung cancer | 0 (0%) | 3.0 (6.3%) | 0.045 |  |

IQR, interquartile range; SMD, standardized mean difference; CEA, carcinoembryonic antigen; CTR, consolidation tumor ratio; SUV, maximum standardized uptake value; LY, lymphatic invasion; V, vascular invasion; PL, pleural invasion; EGFR, epidermal growth factor receptor; STAS, spread through air spaces.
